# Pilot clinical trials testing the safety and effects on the metastatic melanoma microenvironment of intratumoral interferon-gamma or imiquimod, plus a multipeptide melanoma vaccine

**DOI:** 10.1186/2051-1426-3-S2-P139

**Published:** 2015-11-04

**Authors:** Ileana S Mauldin, Nolan A Wages, Anne M Stowman, Ena Wang, Mark E Smolkin, Walter C Olson, Donna H Deacon, Kelly T Smith, Nadejda Galeassi, Jessica E Teague, Rachael A Clark, Francesco M Marincola, Gina R Petroni, David W Mullins, Craig L Slingluff

**Affiliations:** 1 University of Virginia, Charlottesville, VA, USA; 2 Sidra Medical and Research Center, Ar-Rayyan, Qatar; 3 Brigham And Women's Hospital, Boston, MA, USA; 4 Geisel School of Medicine at Dartmouth, Lebanon, NH, USA

## Introduction

A major obstacle to cancer rejection is the failure of T cells to infiltrate and function within the tumor microenvironment (TME). Direct modulation of the TME to promote T cell homing and activation may enhance tumor control. Two pilot clinical trials were conducted to test the hypotheses that either intratumoral interferon-gamma (IFN-γ, Trial A, NCT00977145), or application of a TLR7 agonist, imiquimod (Trial B, NCT01264731) would induce favorable immune signatures and infiltration of CD8^+^ T cells into the TME.

## Methods

Eligible patients received 6 injections of a multipeptide melanoma vaccine and either (Trial A) 2 million units IFN-γ intratumorally once on day 22 (n = 9), or (Trial B) imiquimod applied topically to superficial metastases daily for 6 weeks (n = 4). Melanoma metastases were biopsied pretreatment and at 2 time points during treatment and were evaluated for: immune-cell infiltration by immunohistochemistry, protein expression by Luminex assay, and gene expression by Affymetrix array. T cell responses to vaccination were assessed from peripheral blood by direct *ex vivo* IFN-γ ELIspot assay. Adverse events (CTCAE v4) were recorded.

## Results

One patient experienced grade 3 skin ulceration at a vaccine site (Trial A); otherwise, these combination approaches were well-tolerated. Based on a preliminary analysis, CD8^+^ T cell responses to vaccination were detected in 77% of patients overall. For Trial A, 2 days after IFN-γ, there was an increase in the TME of IFN-inducible chemokine CXCL10 (IP-10), and increased gene expression of the T cell co-stimulatory ligand SECTM1, the immune regulatory enzyme IDO1, and of Complement 4A/4B, and MIR-125B1, but CD8^+^ T cell infiltration of tumors was not increased. In contrast, imiquimod induced dramatic upregulation of immune gene signatures at 3 and 6 weeks; and CD8^+^ T cell infiltration was increased in 75% of patient tumors at 3 weeks.

## Conclusions

These findings support the safety of modulating the TME with intratumoral IFN-γ or topical imiquimod. The data from Trial A are the first to characterize direct effects of IFN-γ on the human melanoma TME, and they suggest possible roles for SECTM1 in mediating T cell co-stimulation, while also supporting prior evidence for induction of the immune checkpoint IDO1. Conversely, imiquimod induced strong immune gene signatures, and promoted CD8^+^ T cell infiltration of tumors. These data support use of imiquimod or other TLR7 agonists in modulating the TME in conjunction with other immune therapies, and highlight the need for better understanding of the processes mediating and limiting T cell infiltration in the TME.

